# Psychiatric disorders associated with fluoroquinolones: a pharmacovigilance analysis of the FDA adverse event reporting system database

**DOI:** 10.3389/fphar.2024.1435923

**Published:** 2024-10-14

**Authors:** Wen-Long Xie, Meng-Lan Ge, Dan Chen, Guo-Qing Chen, Yuan-Xi Mei, Yong-Ji Lai

**Affiliations:** ^1^ Department of Pharmacy, The Central Hospital of Wuhan, Tongji Medical College, Huazhong University of Science and Technology, Wuhan, China; ^2^ Department of Obstetrical, The Central Hospital of Wuhan, Tongji Medical College, Huazhong University of Science and Technology, Wuhan, China; ^3^ Department of Cardiovascular, Changjiang County Integrative Medicine Hospital, Changjiang, China; ^4^ Department of Pharmacology, School of Basic Medicine, Tongji Medical College, Huazhong University of Science and Technology, Wuhan, China

**Keywords:** fluoroquinolones, psychiatric ADRs, pharmacovigilance, FAERS, disproportionality analysis

## Abstract

**Background:**

Fluoroquinolones are broad-spectrum antibiotics with significant antimicrobial activity. Despite their therapeutic benefits, they are associated with a range of adverse drug reactions (ADRs), particularly those affecting the central nervous system (CNS). This study aimed to analyze the psychiatric ADRs linked to fluoroquinolones using data from the FDA Adverse Event Reporting System (FAERS) database.

**Methods:**

A retrospective pharmacovigilance study was conducted using FAERS data from Q1 2004 to Q4 2023. The data processing phase involved the FDA-recommended deduplication method, and ADRs were classified according to Medical Dictionary for Regulatory Activities (MedDRA). Disproportionality analysis was performed using the reporting odds ratio (ROR), and statistical significance was assessed using the Chi-square test or Fisher’s exact test.

**Results:**

The study identified 84,777 reports associated with fluoroquinolones, with 359,480 Preferred Terms-annotated entries, 27,816 of these reports were psychiatric ADRs. Mood disorders were the most frequently reported, including anxiety, depression, and delirium, with some reports escalating to suicidal ideation and behaviors. The Standardized MedDRA Query classification system was used to categorize these ADRs into Depression, Suicide/self-injury, Psychosis and psychotic disorders, and Non-infectious encephalopathy/delirium. Ciprofloxacin was most frequently linked to depression and suicidal ideation, while moxifloxacin showed a robust correlation with delirium. The risk of psychiatric ADRs varied by age group, with affective disorders more prevalent in adults under 65 and psychosis and delirium in those over 65.

**Conclusion:**

Fluoroquinolones are associated with a range of psychiatric ADRs, with notable differences between the drugs in the class. The study highlights the need for caution in prescribing fluoroquinolones, particularly for patients with pre-existing mental health conditions or those in higher risk age groups. The findings also underscore the importance of considering age-specific preventive strategies when administering these antibiotics.

## 1 Introduction

Fluoroquinolones are recognized as a class of broad-spectrum antibiotics that are extensively utilized in clinical practice. They are distinguished by their high oral bioavailability and extensive distribution volume, which contribute to their potent antimicrobial efficacy against both gram-positive and gram-negative bacteria. Consequently, fluoroquinolones are frequently prescribed for a spectrum of infections, including urinary tract infections, pneumonia, sinusitis, tuberculosis, and sexually transmitted diseases (Mahoney and Swords, 2021).

The antimicrobial action of fluoroquinolones is mediated through the inhibition of bacterial DNA gyrase and topoisomerase IV, enzymes essential for DNA replication. While these agents exert a bactericidal effect, they have also been linked to a range of adverse reactions (Leone et al., 2003; Ruan et al., 2024). Notably, central nervous system (CNS)-related adverse drug reactions (ADRs) are the second most frequently reported after gastrointestinal issues and are observed more frequently with fluoroquinolones than with other classes of antimicrobials (Wierzbinski et al., 2023). A statistical review of the literature indicates that the incidence of neuropsychiatric adverse reactions in patients treated with fluoroquinolones can range from 1% to 4.4%, encompassing a spectrum of manifestations from mild symptoms such as confusion, restlessness, and insomnia, to severe conditions including encephalopathy, epileptic seizures, suicidal depression, catatonia, psychosis, and mania (Zareifopoulos and Panayiotakopoulos, 2017). Given the potential for severe side effects, particularly those involving the CNS, the U.S. Food and Drug Administration (FDA) updated the boxed warnings for all oral and injectable fluoroquinolones in 2016 to reflect these risks (Aschenbrenner, 2016).

The CNS effects observed with fluoroquinolones are largely attributed to their high permeability across the blood-brain barrier and their structural resemblance to gamma-aminobutyric acid (GABA). This similarity enables them to interact with GABA(A) receptors in the CNS, thereby competitively inhibiting GABAergic neurotransmission, as discussed in the referenced study (Akaike et al., 1991; Ilgin et al., 2015). Furthermore, the interaction of fluoroquinolones with CNS nicotinic acetylcholine receptors (nAChRs) and N-methyl-D-aspartate receptors (NMDARs) may also significantly contribute to their neuropsychiatric ADRs, as highlighted in the literature (Sanders et al., 2022; Smelt et al., 2018; Zareifopoulos and Panayiotakopoulos, 2017). Additionally, a preclinical study has suggested that the fluoroquinolone ciprofloxacin can induce mitochondrial damage, which could be a critical mechanism underlying CNS injury associated with fluoroquinolone use (Kaur et al., 2016).

The Medicines and Healthcare products Regulatory Agency (MRHA) has recently issued a directive highlighting the elevated incidence of adverse reactions associated with fluoroquinolone use. This directive advises that the prescription of fluoroquinolones be restricted to scenarios where alternative, recommended antibiotics are deemed inappropriate. This follows an earlier safety communication from the MHRA in September 2023, which cautioned about the potential for fluoroquinolones to induce psychiatric reactions, encompassing depression and psychosis, with the possibility of progressing to suicidal ideation or behavior (Author Anonymous, 2024). Given the heightened risk of disability often associated with psychiatric adverse reactions, there has been a growing concern regarding the safety profile of fluoroquinolones. However, a comparative analysis of psychiatric adverse events across the spectrum of fluoroquinolones, including ciprofloxacin, moxifloxacin, and levofloxacin, based on extensive real-world data, remains a relatively unexplored area, with limited systematic studies available to date.

The FDA Adverse Event Reporting System (FAERS) database serves as a pivotal, publicly accessible resource for global pharmacovigilance, compiling adverse event reports from an international perspective, including those within the United States. It is recognized as an authoritative source for the assessment of drug-related ADRs. The current study leverages the FAERS database, extracting data from the first quarter of 2004 through the fourth quarter of 2023, to conduct an in-depth analysis of the ADRs linked to three prevalently prescribed fluoroquinolones: ciprofloxacin, moxifloxacin, and levofloxacin. This research further aims to dissect the psychiatric ADRs attributed to fluoroquinolone use.

## 2 Materials and methods

### 2.1 Data sources

A retrospective pharmacovigilance study was conducted utilizing data extracted from the FAERS database, which is updated quarterly. The FAERS database is a publicly accessible resource that compiles adverse event reports from healthcare professionals, consumers, and other sources globally (Harpaz et al., 2016). For the purpose of this study, reports concerning fluoroquinolones and associated ADRs were systematically collected from the first quarter of 2004 to the fourth quarter of 2023. All data in this study are openly accessed as an ASCII data package from the FAERS website (https://fis.fda.gov/extensions/FPD-QDE-FAERS/FPD-QDE-FAERS.html). Subsequently, the extracted data were processed and in-depth analyzed by Rstudio (Version 2023.06.2 + 561).

### 2.2 Data processing

The data processing phase involved the implementation of the FDA-recommended deduplication method to ensure the reliability of the dataset. Reports with identical CASEID (Number for identifying a FAERS case) in the DEMO (demographics) table were retained based on the highest FDA_DT value. In reports where both CASEID and FDA_DT (date FDA received case) were identical, the report with the maximum PRIMARYID (unique number for identifying a FAERS report) value was preserved. ADRs were classified according to the Medical Dictionary for Regulatory Activities (MedDRA) (Version 26.0). The extraction of data was performed for fluoroquinolones, including ciprofloxacin, moxifloxacin, and levofloxacin, focusing on the System Organ Class (SOC) category of psychiatry-related ADRs. Specific High Level Group Terms (HLGT) relative to mood disorders, as well as Preferred Terms (PT) related to anxiety, depression, suicidal ideation, and other psychiatric conditions were identified and analyzed. The Standardized MedDRA Query (SMQ) was applied to categorize psychiatric ADRs into depression, suicide/self-injury, psychosis, and other psychotic disorders, as well as non-infectious encephalopathy/delirium.

### 2.3 Statistical analysis

The disproportionality analysis was conducted using the reporting odds ratio (ROR) and confidence propagation neural network (BCPNN) methods. The calculation formula is as Supplementary Table S1. ROR ≥1 and 95% CI (lower limit) > 1 with three or more reports considering a positive signal. A higher ROR indicates a stronger correlation between the drug and AE and consequently a stronger risk signal. In the BCPNN analysis method, the lower limit of the 95% confidence interval of the IC (Information Component) value (IC025) is greater than 0, which means that the association between the drug and the adverse event is statistically significant. For the analysis of between-group differences in categorical variables and statistical tests in disproportional analysis, we used the Chi-square test or Fisher’s exact test. P < 0.05 was considered statistically significant (Zhou et al., 2023).

## 3 Result

### 3.1 Descriptive analysis

**FIGURE 1:**
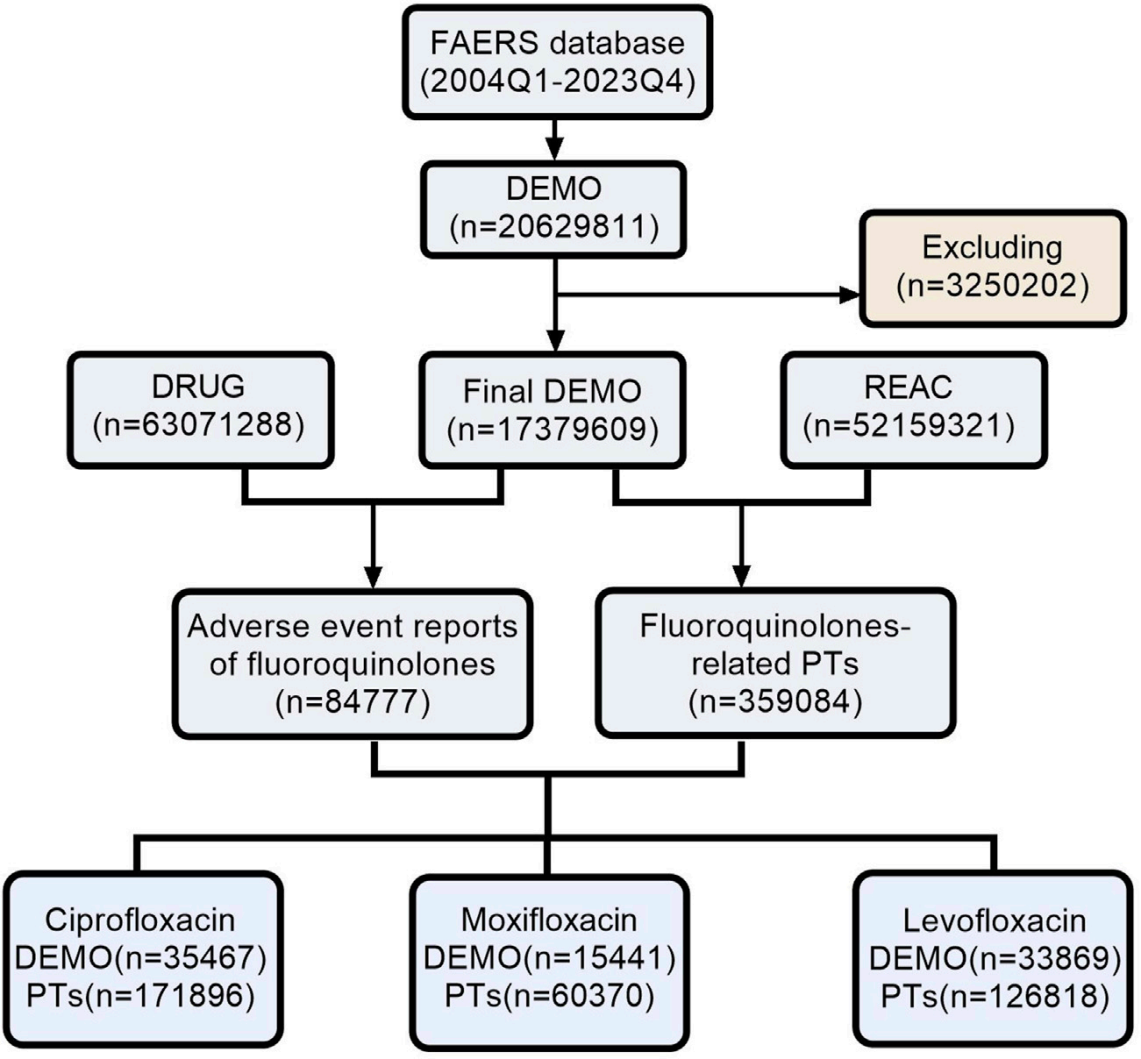
The process of searching fluoroquinolone-associated adverse events from the FAERS. (Abbreviations: FAERS, FDA Adverse Event Reporting System; DEMO, Demographics; REAC, Reactions; PT, Preferred Term).

**FIGURE 2:**
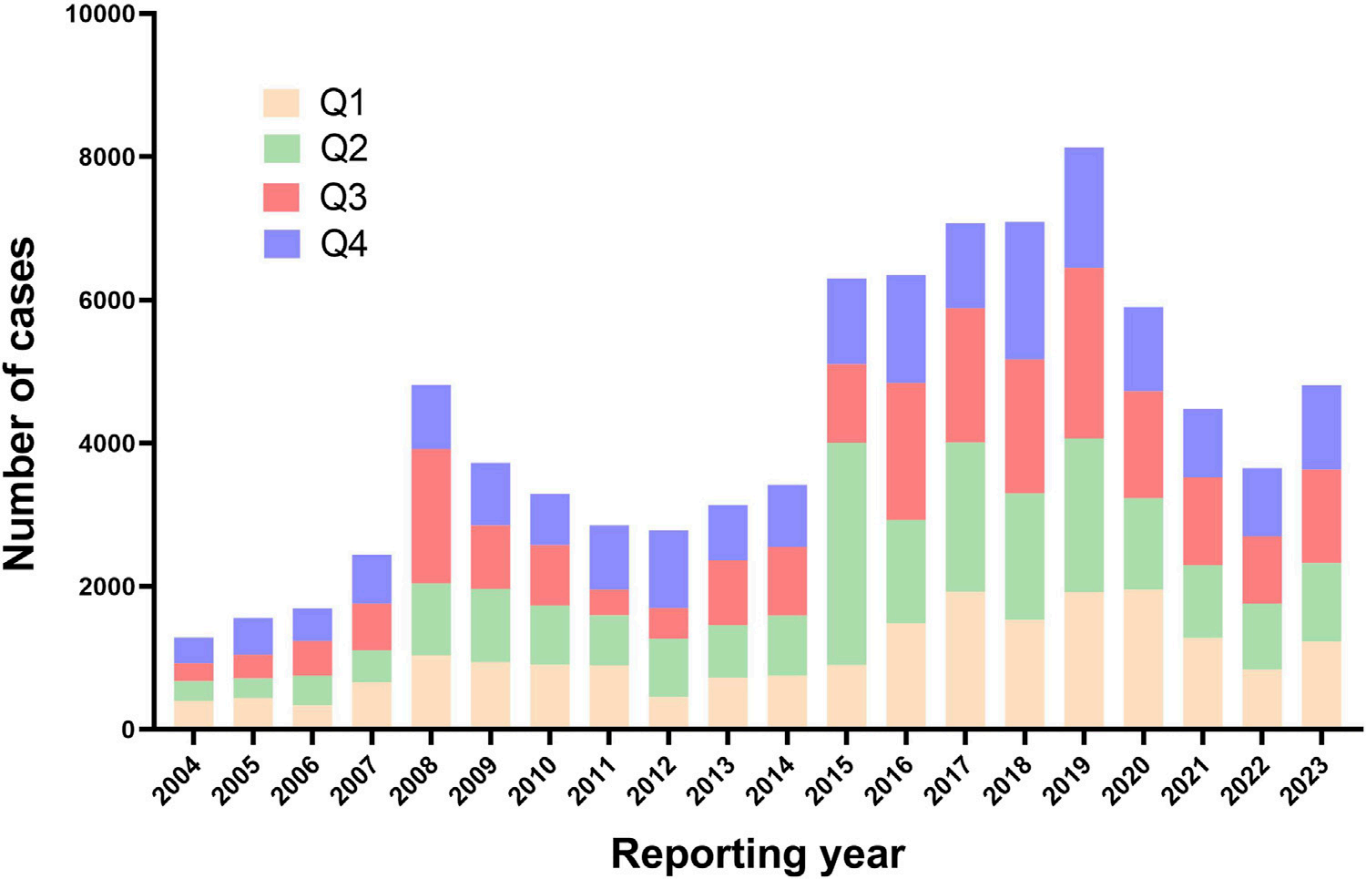
The reporting year of fluoroquinolone-associated adverse events from Q1 2004 to Q4 2023.

As shown in Figure 1, following the removal of duplicate entries, our analysis included a comprehensive dataset of 17, 379, 609 reports from the FAERS, ranging from Q1 2004 to Q4 2023. Specifically, 84,777 reports were linked to fluoroquinolone medications, with detailed breakdowns as follows: Ciprofloxacin (35,467), Moxifloxacin (15,441), and Levofloxacin (33,869). The temporal analysis, as shown in Figure 2, indicates an increased frequency of fluoroquinolone-related ADRs in recent years. Clinical characteristics are outlined in Table 1, with a gender prevalence of females (51.30%) over males (35.20%), a majority of patients within the 50–100 kg weight range (30.30%), and most patients aged 18–64.9 years. The primary sources of reports were Consumers (35.20%) and Physicians (20.60%), with the U.S. contributing 55.60% of reports, followed by the United Kingdom (7.80%), Italy (4.40%), Canada (4.30%), and France (3.60%).

**TABLE 1:** Clinical characteristics of reports with fluoroquinolone from the FAERS database.

Characteristic	Total		Ciprofloxacin		Moxifloxacin		Levofloxacin	
	Case, n	Proportion	Case, n	Proportion	Case, n	Proportion	Case, n	Proportion
Total case	84,777		35,467		15,441		33,869	
Gender								
Female	43,514	51.30%	18,947	0.534	8,075	0.523	16,492	0.487
Male	29,877	35.20%	13,829	0.39	5,091	0.33	10,957	0.324
Missing	11,386	13.40%	2,691	0.076	2,275	0.147	6,420	0.19
Weight								
<50 kg	2,159	2.50%	949	0.027	385	0.025	825	0.024
>100 kg	3,250	3.80%	1,321	0.037	385	0.025	1,544	0.046
50–100 kg	25,679	30.30%	11,547	0.326	3,404	0.22	10,728	0.317
Missing	53,689	63.30%	21,650	0.61	11,267	0.73	20,772	0.613
Age								
<18	1,334	1.60%	812	0.023	151	0.01	371	0.011
>85	2,927	3.50%	1,272	0.036	480	0.031	1,175	0.035
18–64.9	34,246	40.40%	16,073	0.453	5,639	0.365	12,534	0.37
65–85	19,030	22.40%	8,665	0.244	2,820	0.183	7,545	0.223
Missing	27,240	32.10%	8,645	0.244	6,351	0.411	12,244	0.362
Reporter’s type								
Consumer	29,847	35.20%	12,525	0.353	3,371	0.218	13,951	0.412
Physician	17,425	20.60%	5,995	0.169	4,709	0.305	6,721	0.198
Other health-professional	22,562	26.60%	10,408	0.293	4,762	0.309	7,392	0.219
Pharmacist	6,557	7.70%	2,761	0.078	1,210	0.078	2,586	0.076
Lawyer	917	1.10%	442	0.012	226	0.015	249	0.007
Missing	7,469	8.80%	3,336	0.094	1,163	0.075	2,970	0.088
Serious outcome								
Hospitalization—initial or prolonged	22,136	21.10%	9,685	0.216	3,458	0.188	8,993	0.216
Disability	10,357	9.90%	5,456	0.122	706	0.038	4,195	0.101
Life-threatening	5,071	4.80%	2,128	0.047	1,228	0.067	1,715	0.041
Death	3,590	3.40%	1,608	0.036	705	0.038	1,277	0.031
Required intervention to prevent permanent impairment/damage	2,256	2.20%	661	0.015	265	0.014	1,330	0.032
Congenital anomaly	146	0.10%	87	0.002	31	0.002	28	0.001
Other serious	43,765	41.70%	17,958	0.4	7,223	0.393	18,584	0.447
Reported countries (top five)								
America	47,100	55.60%	15,713	0.443	9,565	0.619	21,822	0.644
Britain	7,537	8.90%	6,214	0.176	220	0.014	1,103	0.033
Canada	4,053	4.80%	2,679	0.076	807	0.052	567	0.017
Italy	3,980	4.70%	1,235	0.035	110	0.007	2,635	0.077
France	3,353	4.00%	1,495	0.042	245	0.016	1,613	0.048

### 3.2 Disproportionality analysis

#### 3.2.1 Signal at the SOC level

**FIGURE 3:**
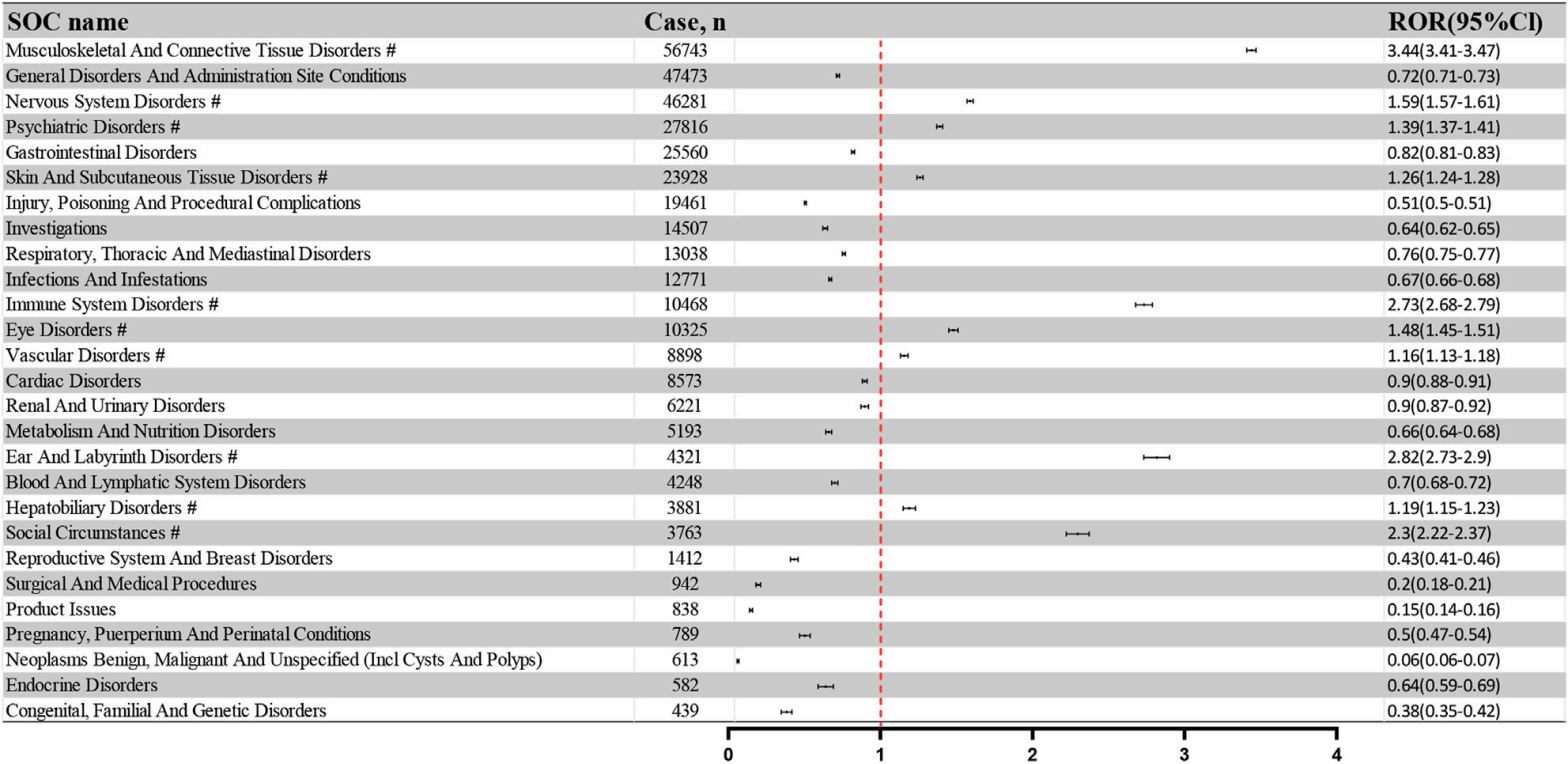
Signal strength of ADRs of fluoroquinolone at the SOC level in FAERS database.

The 359,480 PT-annotated ADRs within the 84,777 adverse event reports were categorized by the System Organ Class (SOC). Figure 3 illustrates the frequency and intensity of these PTs, with significant signals marked by “#.” The top five SOCs by frequency were: Musculoskeletal And Connective Tissue Disorders (56,743 reports; 15.80%), General Disorders And Administration Site Conditions (47,473 reports; 13.22%), Nervous System Disorders (46,281 reports; 12.89%), Psychiatric Disorders (27,816 reports; 7.75%), and Gastrointestinal Disorders (25,560 reports; 7.12%). The corresponding RORs and 95% CIs for these SOCs are as follows: Musculoskeletal And Connective Tissue Disorders (ROR: 3.44, 95%CI: 3.41–3.47), General Disorders And Administration Site Conditions (ROR: 0.72, 95%CI: 0.71–0.73), Nervous System Disorders (ROR: 1.59, 95%CI: 1.57–1.61), Psychiatric Disorders (ROR: 1.39, 95%CI: 1.37–1.41), and Gastrointestinal Disorders (ROR: 0.82, 95%CI: 0.81–0.83).

**FIGURE 4:**
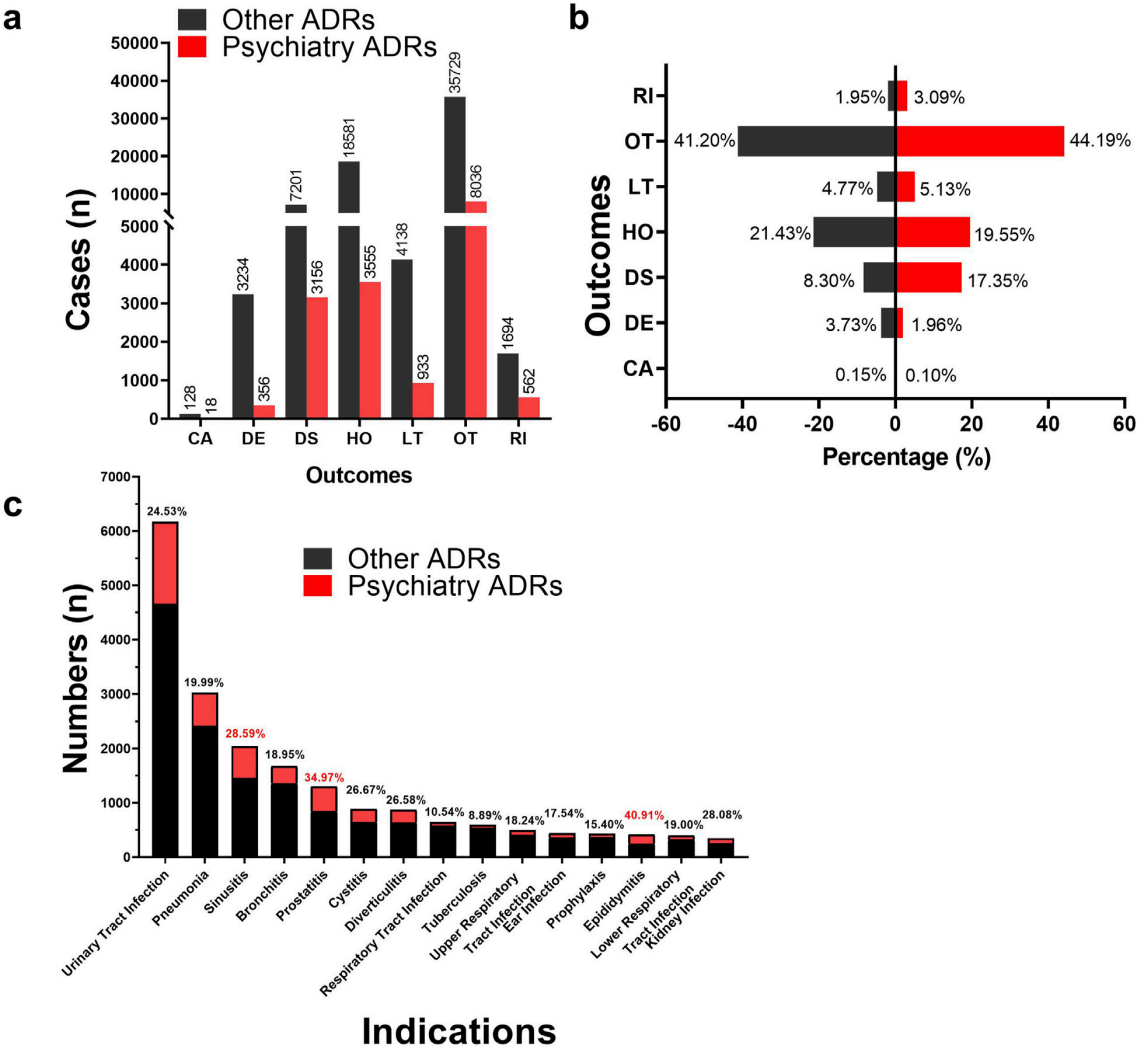
Differences in outcomes and indications between psychiatric ADRs and other ADRs. **(A, B)** Histogram of the difference in frequency and percentage of outcome information reports for psychiatric ADRs and other ADRs. **(C)** Histogram of differences in indications for psychiatric ADRs and other ADRs. (Abbreviations: RI, Required intervention to prevent permanent impairment/damage; OT, Other serious; LT, Life-threatening; HO, Hospitalization—initial or prolonged; DS, Disability; DE, Death; CA, Congenital anomaly).

Figure 4A presents a comparative analysis of recorded outcomes for all ADRs, categorizing them into “Psychiatric ADRs” and “Other ADRs.” Statistical adverse event outcomes include Required intervention to prevent permanent impairment/damage (RI), Life-threatening (LT), Hospitalization—initial or prolonged (HO), Disability (DS), Death (DE), Congenital anomaly (CA), Other serious (OT). As shown in Figure 4B, the Psychiatry ADRs demonstrated a higher proportion of DS outcomes (17.35%) compared to the Other ADRs group (8.30%). A categorical analysis of fluoroquinolone indications, detailed in Figure 4C, reveals the top ten indications by reported ADR case volume. Notably, Epididymitis (40.91%), Prostatitis (34.97%), and Sinusitis (28.59%) showed the highest proportions of psychiatric ADRs.

#### 3.2.2 Signal at the HLGT and PT level

**FIGURE 5:**
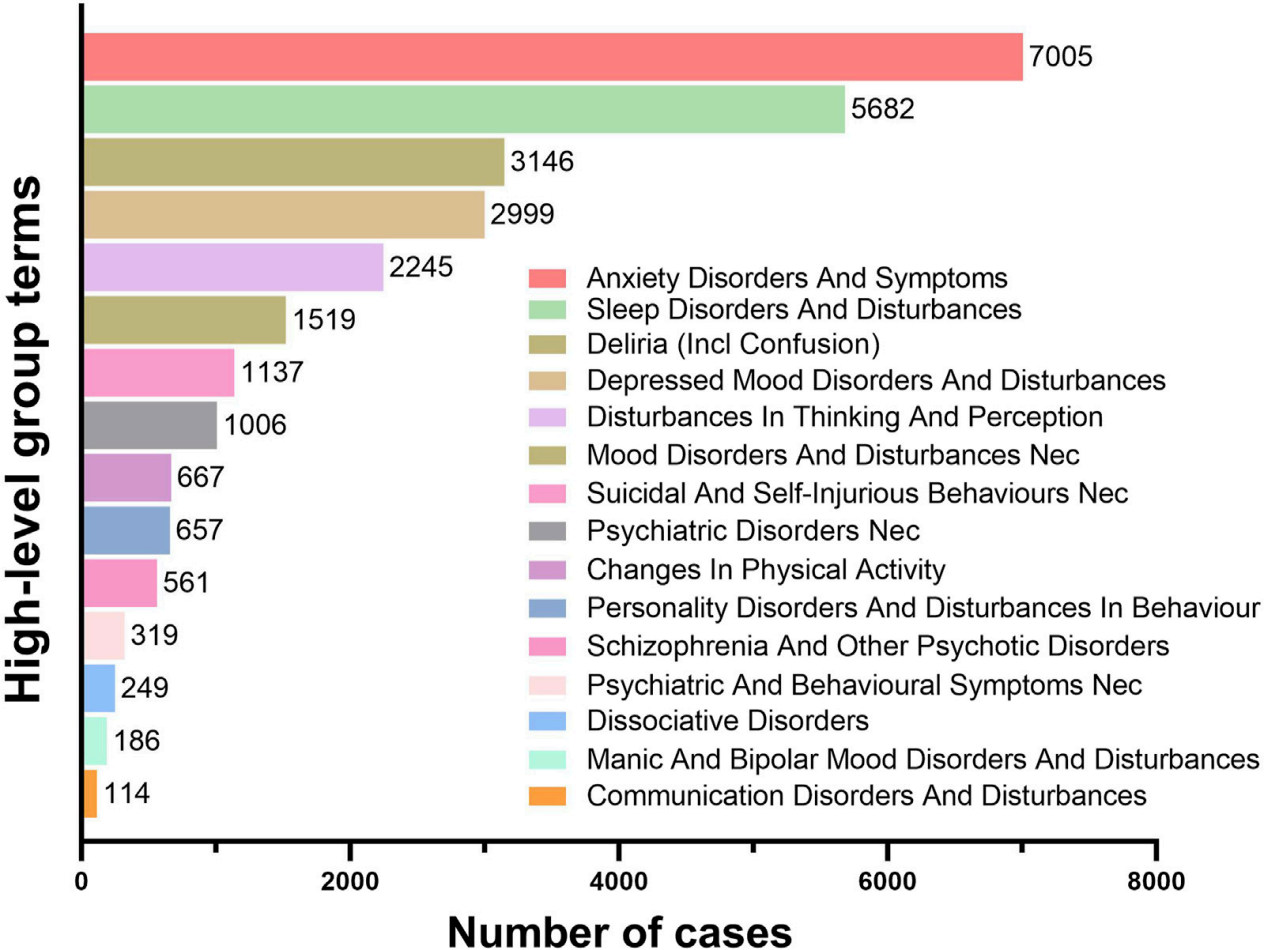
Signal strength of ADRs of fluoroquinolone at the HLGT level in FAERS database.

The PTs under the SOC of Psychiatric Disorders were systematically classified using High-Level Group Terms (HLGTs). Figure 5 lists the top five HLGTs by reporting frequency: Anxiety Disorders And Symptoms (7,005 reports; 25.18%), Sleep Disorders And Disturbances (5,682 reports; 20.43%), Deliria (Including Confusion) (3,146 reports; 11.31%), Depressed Mood Disorders And Disturbances (2,999 reports; 10.78%), and Disturbances In Thinking And Perception (2,245 reports; 8.07%). Table 2 provides the top five PTs for each HLGT, with their ROR (95%Cl) and IC(IC025). The compilation of the most frequently reported PTs reveals that Anxiety (3,680 reports; ROR: 2.17; 95%CI: 2.10–2.25), Insomnia (3,489 reports; ROR: 2.22; 95%CI: 2.15–2.30), Depression (2,353 reports; ROR: 1.70; 95%CI: 1.63–1.77), Confusional State (1,949 reports; ROR: 2.05; 95%CI: 1.96–2.14), and Hallucination (1,089 reports; ROR: 2.58; 95%CI: 2.43–2.74) are at the forefront. Additionally, conditions such as Panic Attack (1,064 reports; ROR: 5.11; 95%CI: 4.80–5.43), Suicidal Ideation (853 reports; ROR: 1.58; 95%CI: 1.48–1.69), Sleep Disorder (808 reports; ROR: 2.08; 95%CI: 1.94–2.23), Agitation (658 reports; ROR: 1.47; 95%CI: 1.36–1.59), and Delirium (622 reports; ROR: 3.23; 95%CI: 2.98–3.50) are also captured within the top ten most frequently reported PTs.

**TABLE 2:** Signal strength of ADRs of the HLGTs-PTs belongs to psychiatric disorders.

HLGTs	PTs (Top five for each hlgt)	Cases, n	ROR (95% Cl)	IC (IC025)
Anxiety Disorders And Symptoms	Anxiety	3,680	2.17 (2.1–2.25)	1.1 (−0.57)
	Panic Attack	1,064	5.11 (4.8–5.43)	2.31 (0.64)
	Agitation	658	1.47 (1.36–1.59)	0.55 (−1.12)
	Nervousness	484	1.49 (1.36–1.63)	0.57 (−1.1)
	Fear	271	1.59 (1.41–1.79)	0.66 (-1.01)
Sleep Disorders And Disturbances	Insomnia	3,489	2.22 (2.15–2.3)	1.13 (−0.53)
	Sleep Disorder	808	2.08 (1.94–2.23)	1.04 (−0.62)
	Nightmare	610	2.95 (2.73–3.2)	1.54 (−0.12)
	Middle Insomnia	196	1.86 (1.61–2.14)	0.89 (−0.78)
	Abnormal Dreams	189	1.1 (0.95–1.27)	0.14 (−1.53)
Deliria (Incl Confusion)	Confusional State	1,949	2.05 (1.96–2.14)	1.02 (−0.64)
	Delirium	622	3.23 (2.98–3.5)	1.67 (0)
	Disorientation	575	2.4 (2.21–2.6)	1.25 (−0.42)
Depressed Mood Disorders And Disturbances	Depression	2,353	1.7 (1.63–1.77)	0.75 (−0.91)
	Depressed Mood	289	0.96 (0.85–1.08)	−0.06 (−1.73)
	Anhedonia	90	0.52 (0.42–0.64)	−0.95 (−2.61)
	Feeling Of Despair	58	2.18 (1.68–2.83)	1.11 (−0.55)
	Depression Suicidal	43	2.07 (1.53–2.8)	1.04 (−0.63)
Disturbances In Thinking And Perception	Hallucination	1,089	2.58 (2.43–2.74)	1.35 (−0.32)
	Hallucination, Visual	296	2.54 (2.26–2.85)	1.33 (−0.34)
	Thinking Abnormal	252	2.2 (1.94–2.49)	1.12 (−0.54)
	Hallucination, Auditory	121	1.3 (1.09–1.56)	0.38 (−1.29)
	Delusion	121	1.35 (1.13–1.62)	0.43 (−1.23)
Mood Disorders And Disturbances Nec	Emotional Distress	319	0.4 (0.36–0.45)	−1.3 (−2.97)
	Irritability	236	0.64 (0.57–0.73)	−0.63 (−2.3)
	Emotional Disorder	146	0.78 (0.67–0.92)	−0.35 (−2.02)
	Mood Altered	145	0.91 (0.77–1.07)	−0.13 (−1.8)
	Anger	132	0.63 (0.53–0.75)	−0.66 (−2.32)
Suicidal And Self-Injurious Behaviours Nec	Suicidal Ideation	853	1.58 (1.48–1.69)	0.66 (−1.01)
	Completed Suicide	105	0.21 (0.17–0.25)	−2.28 (−3.94)
	Suicide Attempt	86	0.24 (0.19–0.29)	−2.06 (−3.72)
	Self-Injurious Ideation	35	1.45 (1.04–2.02)	0.53 (−1.14)
	Suicidal Behaviour	33	1.3 (0.92–1.83)	0.38 (−1.29)
Psychiatric Disorders Nec	Mental Disorder	597	2.35 (2.17–2.55)	1.22 (−0.45)
	Mental Status Changes	186	1.12 (0.97–1.3)	0.17 (−1.5)
	Neuropsychological Symptoms	79	10 (7.96–12.56)	3.23 (1.57)
	Drug Abuse	78	0.16 (0.13–0.2)	−2.66 (−4.33)
	Drug Dependence	10	0.01 (0.01–0.02)	−6.73 (−8.4)
Changes In Physical Activity	Restlessness	551	2.54 (2.34–2.76)	1.33 (−0.34)
	Catatonia	51	1.77 (1.34–2.33)	0.81 (−0.85)
	Tic	33	0.99 (0.7–1.4)	−0.01 (−1.68)
	Bruxism	9	0.28 (0.15–0.55)	−1.81 (−3.48)
	Head Banging	5	1.47 (0.61–3.55)	0.55 (−1.12)
Personality Disorders And Disturbances In Behaviour	Paranoia	270	2.62 (2.32–2.96)	1.37 (−0.29)
	Aggression	204	0.67 (0.58–0.77)	−0.58 (−2.25)
	Personality Change	54	0.93 (0.71–1.21)	−0.11 (−1.78)
	Social Avoidant Behaviour	28	0.76 (0.52–1.1)	−0.39 (−2.06)
	Violence-Related Symptom	19	1.95 (1.24–3.06)	0.95 (−0.72)

Abbreviations: HLGT, high level group terms; PT, preferred terms; ROR, reporting odds ratio; CI, confidence interval; Nec, not elsewhere classified; Incl, inclusion; IC, information component.

Supplementary Figure S1 utilizes heat maps to depict the reporting frequency and ROR values for Ciprofloxacin, Moxifloxacin, and Levofloxacin, highlighting variations in ADRs profiles. To address potential confounding factors in spontaneous reporting databases, we performed subgroup analyses. As shown in Supplementary Figures S2, S3, we subgrouped according to gender and age group and showed the top 20 PTs in terms of frequency of reporting for Females and Males, and the top 10 PTs in terms of frequency of reporting for each age group.

### 3.3 SMQ analysis

**FIGURE 6:**
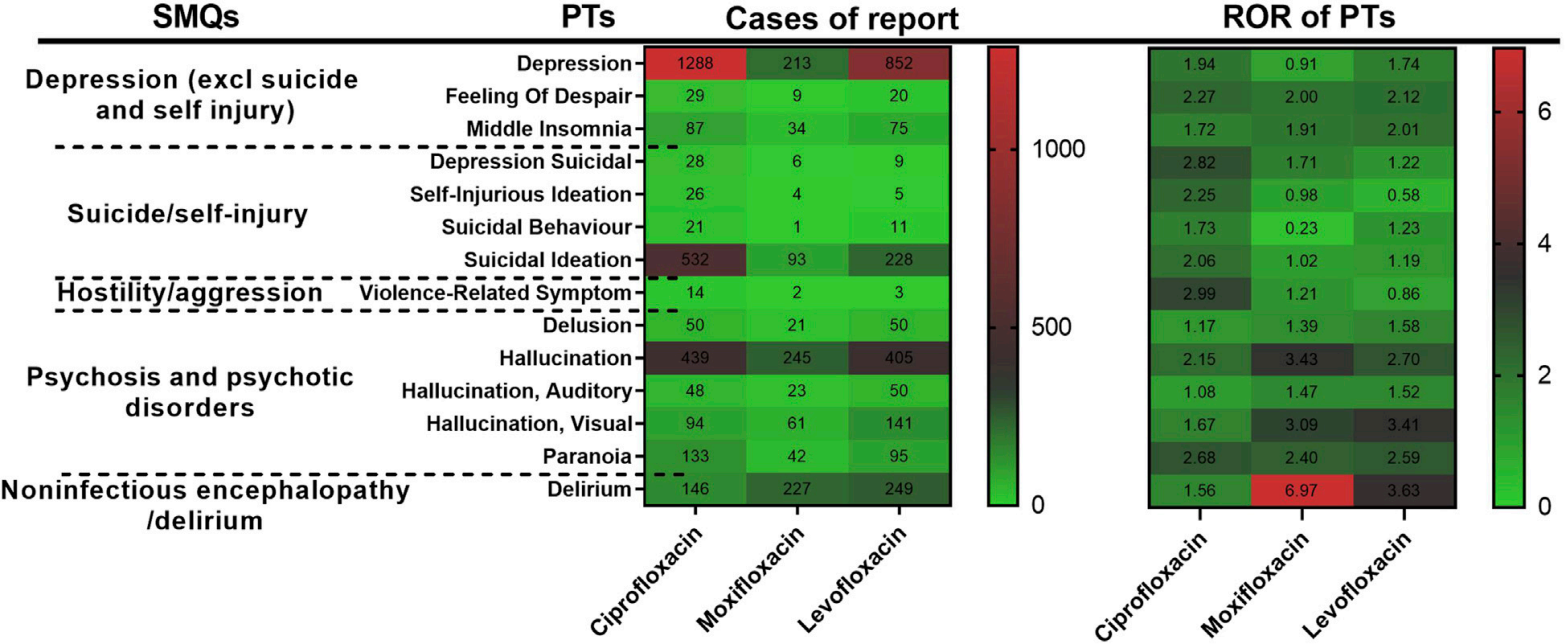
Heatmap of signal strength of PTs after SMQ clustering.

SMQs are utilized to focus on specific medical conditions using curated MedDRA terms, including PTs for signs, symptoms, diagnoses, and more. SMQs can be broad or narrow in scope, with the latter chosen for this study to enhance specificity. All PTs for psychiatric ADRs with positive signal were screened and categorized into narrow SMQs. Figure 6 classifies these reactions into five SMQs: Depression (excluding suicide and self-injury), Suicide/Self-Injury, Hostility/Aggression, Psychosis and Psychotic Disorders, and Noninfectious Encephalopathy/Delirium, with the heat map illustrating their frequency and ROR values. The IC (IC025) values of the three fluoroquinolones for each PT are displayed in Supplementary Table S2. Hostility/Aggression, due to its low report frequency, was not further analyzed.

Table 3 outlines the general characteristics of the four remaining SMQs and analyzes these variables for significance using the Chi-square test or Fisher’s exact test, excluding missing data. Notable differences in gender and age distribution were found for the Depression SMQ (p < 0.05), with a higher proportion of females and concentration in the 18–64.9 age group. The suicide/self-injury age distribution also significantly differed, with 71.2% of cases in the 18–64.9 age group. Psychosis and psychotic disorders showed a significant distribution related to weight and age, with higher frequencies in the underweight and over 85 years groups. Noninfectious encephalopathy/delirium PTs were more often reported in males and underweight individuals, with a higher proportion in those over 65 years.

**TABLE 3:** Clinical characteristics of reports with fluoroquinolone at the SMQ level in FAERS database.

SMQs	Clinical characteristics	NO_target PTs	Target PTs	*p*-value
Depression (excl suicide and self injury) (DEMO, n = 2,559)	Gender			0.0486
	F	42,023 (51.1%)	1,491 (58.3%)	
	M	28,933 (35.2%)	944 (36.9%)	
	Missing	11,262 (13.7%)	124 (4.8%)	
	Weight			0.1475
	<50 kg	2,033 (2.5%)	126 (4.9%)	
	>100 kg	3,091 (3.8%)	159 (6.2%)	
	50–100 kg	24,212 (29.4%)	1,467 (57.3%)	
	Missing	52,882 (64.3%)	807 (31.5%)	
	Age			<0.0001
	<18	1,322 (1.6%)	12 (0.5%)	
	>85	2,910 (3.5%)	17 (0.7%)	
	18–64.9	32,496 (39.5%)	1750 (68.4%)	
	65–85	18,724 (22.8%)	306 (12.0%)	
	Missing	26,766 (32.6%)	474 (18.5%)	
Suicide/self-injury (DEMO, n = 931)	Gender			0.2262
	F	43,013 (51.3%)	501 (53.8%)	
	M	29,502 (35.2%)	375 (40.3%)	
	Missing	11,331 (13.5%)	55 (5.9%)	
	Weight			0.6502
	<50 kg	2,120 (2.5%)	39 (4.2%)	
	>100 kg	3,188 (3.8%)	62 (6.7%)	
	50–100 kg	25,152 (30.0%)	527 (56.6%)	
	Missing	53,386 (63.7%)	303 (32.5%)	
	Age			<0.0001
	<18	1,330 (1.6%)	4 (0.4%)	
	>85	2,923 (3.5%)	4 (0.4%)	
	18–64.9	33,583 (40.1%)	663 (71.2%)	
	65–85	18,924 (22.6%)	106 (11.4%)	
	Missing	27,086 (32.3%)	154 (16.5%)	
Psychosis and psychotic disorders (DEMO, n = 1,696)	Gender			0.0607
	F	42,553 (51.2%)	961 (56.7%)	
	M	29,278 (35.2%)	599 (35.3%)	
	Missing	11,250 (13.5%)	136 (8.0%)	
	Weight			0.0002
	<50 kg	2,064 (2.5%)	95 (5.6%)	
	>100 kg	3,167 (3.8%)	83 (4.9%)	
	50–100 kg	24,935 (30.0%)	744 (43.9%)	
	Missing	52,915 (63.7%)	774 (45.6%)	
	Age			<0.0001
	<18	1,307 (1.6%)	27 (1.6%)	
	>85	2,784 (3.4%)	143 (8.4%)	
	18–64.9	33,503 (40.3%)	743 (43.8%)	
	65–85	18,581 (22.4%)	449 (26.5%)	
	Missing	26,906 (32.4%)	334 (19.7%)	
Noninfectious encephalopathy/delirium (DEMO, n = 622)	Gender			<0.0001
	F	43,261 (51.4%)	253 (40.7%)	
	M	29,555 (35.1%)	322 (51.8%)	
	Missing	11,339 (13.5%)	47 (7.6%)	
	Weight			<0.0001
	<50 kg	2,132 (2.5%)	27 (4.3%)	
	>100 kg	3,242 (3.9%)	8 (1.3%)	
	50–100 kg	25,446 (30.2%)	233 (37.5%)	
	Missing	53,335 (63.4%)	354 (56.9%)	
	Age			<0.0001
	<18	1,321 (1.6%)	13 (2.1%)	
	>85	2,829 (3.4%)	98 (15.8%)	
	18–64.9	34,085 (40.5%)	161 (25.9%)	
	65–85	18,761 (22.3%)	269 (43.2%)	
	Missing	27,159 (32.3%)	81 (13.0%)	

**FIGURE 7:**
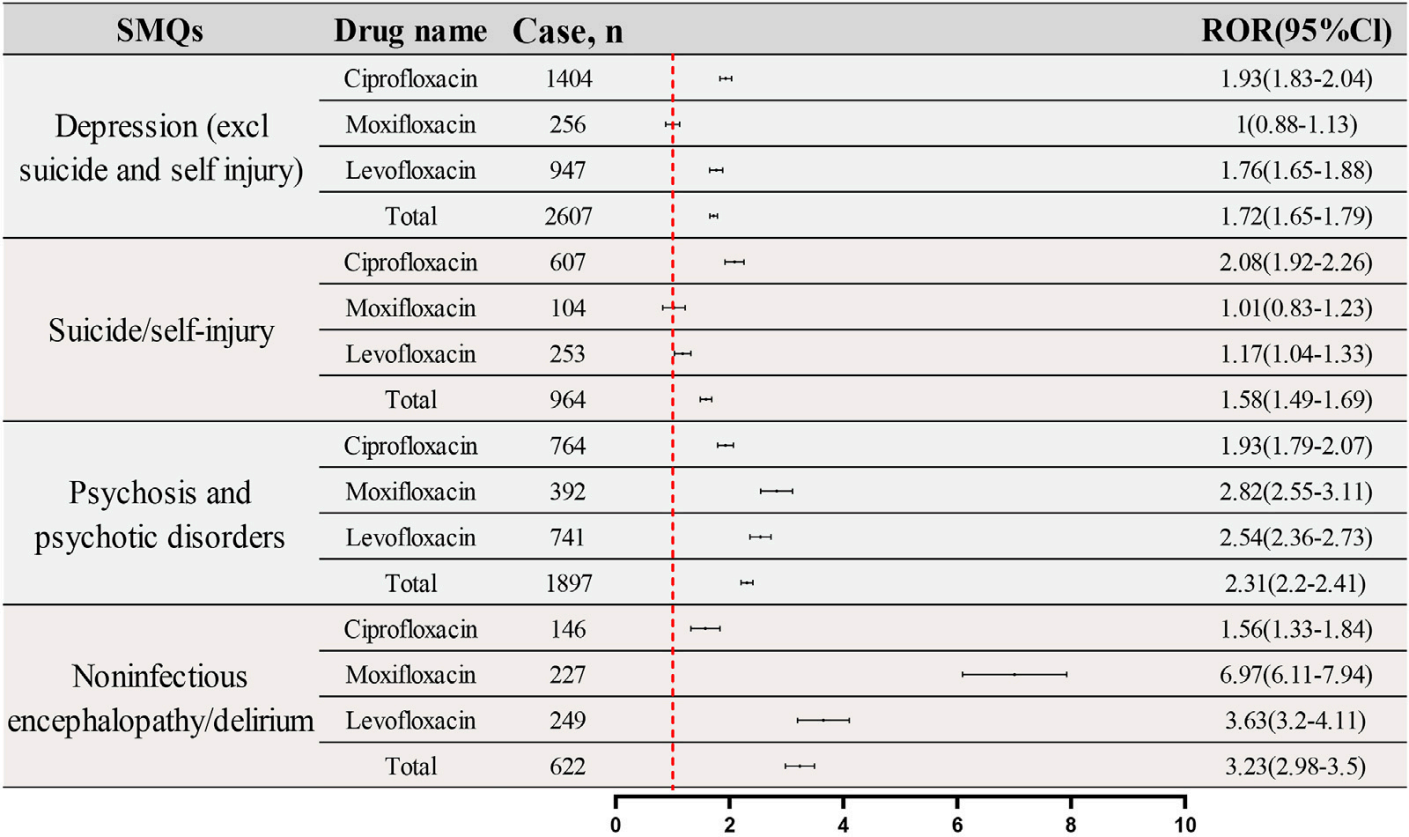
Signal strength of ADRs of fluoroquinolones at the SMQ level in FAERS database.

Figure 7 presents a comparative analysis of the reporting frequencies and RORs for four specified SMQs across three fluoroquinolone drugs: Ciprofloxacin, Moxifloxacin, and Levofloxacin. The analysis provides a detailed examination of the ADRs profiles associated with each medication. For the SMQ “Depression (excluding suicide and self-injury),” the reported reports and RORs are as follows: Ciprofloxacin (1,404 reports; ROR: 1.93; 95%CI: 1.83–2.04), Moxifloxacin (256 reports; ROR: 1.00; 95%CI: 0.88–1.13), and Levofloxacin (947 reports; ROR: 1.76; 95%CI: 1.65–1.79). In the “Suicide/Self-Injury” category, the reported reports and RORs are: Ciprofloxacin (607 reports; ROR: 2.08; 95%CI: 1.92–2.26), Moxifloxacin (104 reports; ROR: 1.01; 95%CI: 0.83–1.23), and Levofloxacin (253 reports; ROR: 1.17; 95%CI: 1.04–1.33). The IC (IC025) values of the three fluoroquinolones for each SMQ are displayed in Supplementary Table S3.

The SMQ “Psychosis and Psychotic Disorders” reveals the following reported reports and RORs: Ciprofloxacin (764 reports; ROR: 1.93; 95%CI: 1.79–2.07), Moxifloxacin (392 reports; ROR: 2.82; 95%CI: 2.55–3.11), and Levofloxacin (741 reports; ROR: 2.54; 95%CI: 2.36–2.73). Lastly, for “Noninfectious Encephalopathy/Delirium,” the reported reports and RORs are: Ciprofloxacin (146 reports; ROR: 1.56; 95%CI: 1.33–1.84), Moxifloxacin (227 reports; ROR: 6.97; 95%CI: 6.11–7.94), and Levofloxacin (249 reports; ROR: 3.63; 95%CI: 3.20–4.11). These findings offer a nuanced perspective on the differential reporting patterns and signal intensities of psychiatric ADRs related to fluoroquinolone drugs, as identified by the respective SMQs.

## 4 Discussion

In the present study, we undertook a comprehensive mining and analysis of adverse event reports for fluoroquinolones, as documented in the FAERS database. Our focus was on events categorized under the SOC pertaining to mental disorders. Notably, within the HLGT designations of ADRs associated with fluoroquinolones, mood disorders were predominantly reported. This includes a spectrum of conditions such as anxiety disorders and major depressive episodes, alongside other significant psychiatric disorders like delirium. In severe instances, these events extended to manifestations of suicidal ideation or the occurrence of suicidal behaviors. SMQ classification further delineated the psychiatric ADRs into specific categories, encompassing depression, suicide/self-injury, psychosis and other psychotic disorders, and non-infectious encephalopathy/delirium. Further analysis revealed that the risk of psychiatric ADRs varied by age group, with affective disorders more prevalent in adults under 65 and psychosis and delirium in those over 65. These findings collectively imply that the prescribing and utilization of fluoroquinolones should be approached with an awareness of the potential risks to mental health.

We systematically analyzes the psychiatric adverse events associated with fluoroquinolone drugs in the FAERS database, which shows both similarities and differences with many existing literature results. In a systematic review, a meta-analysis of psychiatric adverse reactions reported from 68 pieces of literature on fluoroquinolone drugs was conducted, which, consistent with this research, also reported adverse reactions such as delirium, psychosis, hallucinations, insomnia, and depression caused by fluoroquinolones (Wierzbinski et al., 2023). However, due to the small sample size of this paper, the statistics on the types of psychiatric adverse reactions are not as comprehensive as this study, and the impact of factors such as age and gender on the risk of adverse events cannot be assessed. A population-based cohort study in Ontario, Canada, showed that elderly patients using high doses of fluoroquinolones were more likely to experience psychiatric adverse reactions, including disturbances of consciousness such as delirium, disorientation, temporary changes in consciousness, agitation, and tension (Muanda et al., 2022). HARTINGER et al. also pointed out in a retrospective study that psychiatric adverse reactions caused by ciprofloxacin are common in elderly patients over 70 years old, with an incidence rate as high as 13.6% in the group over 80 years old (Hartinger et al., 2023). In this study, we found that in the elderly group over 65 years old, psychosis and delirium are more common adverse reactions, while the group under 65 years old is more prone to emotional disorders. This study took advantage of the large volume of data in the FAERS database for a more detailed analysis, and the study evaluated the correlation between adverse events and drugs through disassociation analysis, clearly reflecting the degree of correlation.

Within the spectrum of psychiatric ADRs induced by fluoroquinolones, a substantial proportion was attributed to Anxiety Disorders And Symptoms, with Anxiety and Panic Attack being particularly prevalent in terms of frequency and severity. While anxiety disorders are typically associated with mild outcomes that do not impede the course of treatment, they are infrequently linked to fluoroquinolone use. Nonetheless, evidence of fluoroquinolone-induced anxiety is emerging from both preclinical and clinical studies. Experimental data demonstrate that enrofloxacin can elicit anxiety-like behaviors in zebrafish via modulation of the gut-brain axis (Tian et al., 2023), and sparfloxacin has been observed to elevate anxiety levels in murine models (Bharal et al., 2006). Furthermore, oral administration of ciprofloxacin and norfloxacin has been documented to induce anxiogenic effects in rats (Sen et al., 2007). Clinical reports have also described anxiety disorders associated with the use of ciprofloxacin and levofloxacin (Maharani et al., 2019; Munjal and Smolin, 2017; Kandasamy and Srinath, 2012). In our analysis, anxiety emerged as a common ADRs, and ciprofloxacin was more frequently associated with anxiety adverse events relative to moxifloxacin and levofloxacin. Given that fluoroquinolones are known to competitively bind to the GABA-A receptor (GABA_A_R) (Halliwell et al., 1995; Akaike et al., 1991), a mechanism that typically serves an anxiolytic function, the pathogenesis of anxiety symptoms induced by these agents may be related to their inhibitory effects on GABAergic neurotransmission. Additionally, benzodiazepines, known for their sedative properties, act through the GABA system (Kim and Hibbs, 2021; Miczek et al., 1995), further underscoring the significance of this pathway in anxiety modulation. Conversely, the impact of fluoroquinolones on gut microbiota may also contribute to anxiety symptoms, as perturbations in the gut microbiota can influence anxiety levels via the gut-brain axis (Tian et al., 2023). It is conceivable that the heightened affinity of ciprofloxacin for GABA_A_R, in conjunction with the sensitivity of certain gut microbiota to anxiety, may precipitate a higher likelihood of anxiety-related ADRs with this particular fluoroquinolone.

Clinical case reports have detailed instances of delirium induced by moxifloxacin in the geriatric population, highlighting the neuropsychiatric risks associated with this class of antibiotics (Pozo et al., 2015). Furthermore, there is documentation of levofloxacin-triggered delirium in a patient with comorbid schizophrenia and multiple sclerosis (Lertxundi et al., 2013). A retrospective analysis of 631 veterans exposed to fluoroquinolones revealed a notable rate of psychosis/delirium, approximately 3.7%, with ciprofloxacin and moxifloxacin being more strongly associated with delirium than levofloxacin (Sellick et al., 2018). While delirium as an ADR to quinolones is sometimes underappreciated, our study, employing a disproportionality analysis method, has identified a robust correlation between fluoroquinolone use and the incidence of delirium, with a particularly significant association observed for moxifloxacin. The data presented in Table 3 of our study further indicate that the frequency of reported delirium was significantly higher in those aged 65–85 years and those aged 85 years and above, suggesting that increased age is a potential risk factor for this adverse event. Additionally, our findings suggest that delirium is reported more frequently in men compared to women. These observations imply that a heightened index of suspicion and preventive measures may be warranted when administering moxifloxacin to elderly male patients to mitigate the risk of delirium.

Despite the number of reports of depression- and suicide-related ADRs associated with fluoroquinolone antibiotics, the scrutiny accorded to this phenomenon has been relatively low, with a dearth of studies specifically addressing the issue. A case report documented the onset of suicidal ideation in a 75-year-old subject following treatment with levofloxacin (Lasalvia et al., 2010). Another case study revealed the emergence of depressive symptoms and suicidal ideation in a 26-year-old patient upon sequential exposure to ciprofloxacin, subsequent to a combination therapy involving levofloxacin and metronidazole (Labay-Kamara et al., 2012). A review of the FDA’s adverse event reports linked to fluoroquinolones identified that incidents of suicidal behavior were frequently reported within a fortnight of the commencement of fluoroquinolone therapy (Kommalapati et al., 2018). Notably, even among patients devoid of a psychiatric history, suicidal ideation or attempts have been observed following fluoroquinolone use, with a subsequent resolution of symptoms upon cessation of the medication (Lasalvia et al., 2010; Labay-Kamara et al., 2012; Ahmed et al., 2011). In the current study, the PTs for Depression and Suicidal Ideation exhibited relatively elevated reporting frequencies within the context of ADRs associated with fluoroquinolone antibiotics. Our analysis utilizing the SMQ classification further substantiated the association of psychiatric ADRs of quinolones with Depression and Suicide/Self-Injury categories. Notably, Ciprofloxacin emerged as the fluoroquinolone most frequently implicated in reports of depression and suicidal ideation, with the highest reporting intensity observed among the trio of fluoroquinolones under review. Conversely, Moxifloxacin appeared to be associated with a potentially lower risk of Depression and Suicide/Self-Injury related ADRs. These findings underscore the importance of a nuanced approach when selecting fluoroquinolone therapy, particularly for patients with pre-existing depression or established suicide risk factors. In such cases, Moxifloxacin may be considered the treatment of choice, prioritizing patient safety and mental health considerations.

In the present study, the “Psychosis and Psychotic Disorders” category within the SMQ classification was observed to encompass a significant proportion of hallucination-related ADRs. These included, but were not limited to, “hallucination,” “auditory hallucination,” and “visual hallucination.” Although ciprofloxacin was associated with a relatively elevated frequency of such hallucinatory ADRs, the intensity of reporting for this particular drug was found to be less pronounced in comparison to levofloxacin and moxifloxacin. Conversely, the risk of inducing paranoia was noted to be analogous across the three fluoroquinolone antibiotics under review. The etiology of hallucinatory ADRs attributed to fluoroquinolones may be multifactorial, potentially linked to both the dosage levels of fluoroquinolones and their concomitant use with other pharmacological agents (Bhattacharya et al., 2017; Chauhan et al., 2013). Additionally, the relationship between these ADRs and the patient’s psychiatric history warrants further investigation, with the necessity for corroboration through extensive real-world data analysis.

Fluoroquinolones, when juxtaposed with other antibiotic classes, have been observed to elicit a higher incidence of psychiatric ADRs, particularly in the geriatric population who are more susceptible to neurotoxic symptoms (Mattappalil and Mergenhagen, 2014). However, the validity of this age-related inference is constrained by the limited sample size within the surveyed data corpus. This limitation is partially mitigated by the extensive data repository of the FAERS, which affords a broader perspective on the observed trends. Upon application of the SMQ classification framework, this study discerned significant disparities in the age distribution of subjects experiencing ADRs across four distinct categories, when compared to the general ADRs profile. The primary reported population for depression and suicide/self-injury was identified as the 18–64.9 year age group. In contrast, the proportion of individuals over 65 years of age was disproportionately higher within the Psychosis and Psychotic Disorder and Noninfectious Encephalopathy/Delirium categories. We therefore hypothesized that the incidence of psychiatric adverse effects caused by fluoroquinolones is not invariably elevated in the elderly but rather that affective disorders are more prevalent in adults below the age of 65. It is important to note that the use of fluoroquinolones in the pediatric population is often restricted, which may introduce a degree of bias into the analysis. Nevertheless, the data imply a correlation between the incidence of psychiatric adverse reactions and age, suggesting that different age cohorts may require targeted preventative strategies when administering fluoroquinolones.

FAERS is an essential drug safety monitoring tool, but it has limitations that can affect the interpretation of data on fluoroquinolone antibiotics and their potential to cause psychiatric ADRs. Voluntary reporting can lead to underreporting, especially for conditions like psychiatric disorders that may be misdiagnosed. The dataset may also be biased towards more severe reactions, potentially distorting the prevalence of certain effects. Additionally, FAERS lacks detailed patient information, such as concurrent medications, which could influence the development of psychiatric reactions and obscure the assessment of drug interactions. The database does not offer direct incidence rates, complicating the determination of actual risks associated with fluoroquinolone use. Moreover, the database’s age-related data may not represent the broader population, particularly concerning the elderly who are more susceptible to neurotoxic effects. Importantly, FAERS data cannot establish causation; it only indicates potential associations. The causes of adverse reactions, like hallucinations or suicidal thoughts, are likely multifactorial and require further investigation to establish a definitive link to fluoroquinolones. In conclusion, while FAERS is a valuable resource, its limitations must be acknowledged when interpreting data on psychiatric adverse reactions related to fluoroquinolones. Further research, including prospective studies and real-world data analysis, is essential to confirm and expand upon FAERS findings.

## 5 Conclusion

The study presents a thorough analysis of the FAERS database to investigate the psychiatric adverse events associated with fluoroquinolone antibiotics. The research, focusing on mental disorders, revealed that mood disorders were the most frequently reported, including anxiety, depression, and delirium, with some reports escalating to suicidal ideation and behaviors. The SMQ classification system was used to categorize these ADRs into Depression, Suicide/self-injury, Psychosis and psychotic disorders, and Non-infectious encephalopathy/delirium. Anxiety disorders and symptoms were particularly prevalent, with ciprofloxacin showing a higher anxiogenic potential, possibly due to its interaction with the GABA-A receptor and its impact on gut microbiota. Delirium, especially in the elderly, was significantly associated with fluoroquinolone use, with moxifloxacin showing a robust correlation. The potential for fluoroquinolones to induce suicidal ideation deserves more attention. Hallucination-related ADRs were significant, with a multifactorial etiology potentially involving dosage levels and concomitant medication use. The study found that the frequency of reported psychiatric ADRs varied by age group, with more ADRs related to affective disorders reported in adults under 65 years of age, compared to psychosis and delirium in the group over 65 years of age. The findings emphasize the need for caution in fluoroquinolone prescription and suggest that age-specific preventive strategies may be necessary.

## Data Availability

Publicly available datasets were analyzed in this study. This data can be found here: All data in this study are openly accessed as an ASCII data package from the FAERS website: https://fis.fda.gov/extensions/FPD-QDE-FAERS/FPD-QDE-FAERS.html.

## References

[B1] Ahmed A. I. van der Heijden F. M. van den Berkmortel H. Kramers K. (2011). A man who wanted to commit suicide by hanging himself: an adverse effect of ciprofloxacin. Gen. Hosp. Psych. 33 (1), 82.e5–e7. 10.1016/j.genhosppsych.2010.07.002 21353135

[B2] Akaike N. Shirasaki T. Yakushiji T. (1991). Quinolones and fenbufen interact with GABAA receptor in dissociated hippocampal cells of rat. J. Neurophysiol. 66 (2), 497–504. 10.1152/jn.1991.66.2.497 1723095

[B3] Aschenbrenner D. S. (2016). The FDA revises boxed warning for fluoroquinolones-again. Am. J. Nurs. 116 (9), 22–23. 10.1097/01.NAJ.0000494691.55746.90 27560334

[B4] Author Anonymous (2024). MHRA issues two updates on fluoroquinolone safety. Drug Ther. Bull. 62 (2), 19. 10.1136/dtb.2023.000069 38123946

[B5] Bharal N. Pillai K. K. Vohora D. (2006). Effects of sparfloxacin on CNS functions and urinary hydroxyproline in mice. Pharmacol. Res. 54 (2), 111–117. 10.1016/j.phrs.2006.03.004 16701999

[B6] Bhattacharya A. Sharan R. Praharaj S. K. (2017). High dose ofloxacin-induced bimodal hallucinations in a 4 Years old child. Clin. Psychopharmacol. Neurosci. 15 (4), 416–417. 10.9758/cpn.2017.15.4.416 29073756 PMC5678492

[B7] Chauhan U. Shanbag P. Kashid P. (2013). Ofloxacin-induced hallucinations. Indian J. Pharmacol. 45 (2), 189–190. 10.4103/0253-7613.108316 23716899 PMC3660935

[B8] Halliwell R. F. Davey P. G. Lambert J. J. (1995). A patch clamp study of the effects of ciprofloxacin and biphenyl acetic acid on rat hippocampal neurone GABAA and ionotropic glutamate receptors. Neuropharmacology 34 (12), 1615–1624. 10.1016/0028-3908(95)00106-9 8788959

[B9] Harpaz R. Dumochel W. Shah N. H. (2016). Big data and adverse drug reaction detection. Clin. Pharmacol. Ther. 99 (3), 268–270. 10.1002/cpt.302 26575203

[B10] Hartinger J. M. Dvorackova E. Myslivecek M. Hruskova Z. Satny M. Zlatohlavek L. (2023). The frequency of, and predisposing risk factors for, ciprofloxacin-induced neuro-psychiatric adverse drug reactions. Bratisl. Lek. Listy. 124 (10), 779–782. 10.4149/BLL_2023_120 37789796

[B11] Ilgin S. Can O. D. Atli O. Ucel U. I. Sener E. Guven I. (2015). Ciprofloxacin-induced neurotoxicity: evaluation of possible underlying mechanisms. Toxicol. Mech. Methods. 25 (5), 374–381. 10.3109/15376516.2015.1026008 25902267

[B12] Kandasamy A. Srinath D. (2012). Levofloxacin-induced acute anxiety and insomnia. J. Neurosci. Rural. Pract. 3 (2), 212–214. 10.4103/0976-3147.98256 22865986 PMC3410005

[B13] Kaur K. Fayad R. Saxena A. Frizzell N. Chanda A. Das S. (2016). Fluoroquinolone-related neuropsychiatric and mitochondrial toxicity: a collaborative investigation by scientists and members of a social network. J. Community Support Oncol. 14 (2), 54–65. 10.12788/jcso.0167 26955658

[B14] Kim J. J. Hibbs R. E. (2021). Direct structural insights into GABA(A) receptor pharmacology. Trends biochem. Sci. 46 (6), 502–517. 10.1016/j.tibs.2021.01.011 33674151 PMC8122054

[B15] Kommalapati A. Wallam S. Tella S. H. Qureshi Z. P. Bennett C. L. (2018). Fluoroquinolone-associated suicide. Eur. J. Intern. Med. 55, e21–e22. 10.1016/j.ejim.2018.07.012 30031596

[B16] Labay-Kamara U. Manning S. Mcmahon T. (2012). Fluoroquinolone-induced suicidal ideation and suicidality. Psychosomatics 53 (1), 97–98. 10.1016/j.psym.2011.05.003 22221728

[B17] Lasalvia E. A. Domek G. J. Gitlin D. F. (2010). Fluoroquinolone-induced suicidal ideation. Gen. Hosp. Psych. 32 (1), 108–110. 10.1016/j.genhosppsych.2009.03.002 20114138

[B18] Leone R. Venegoni M. Motola D. Moretti U. Piazzetta V. Cocci A. (2003). Adverse drug reactions related to the use of fluoroquinolone antimicrobials: an analysis of spontaneous reports and fluoroquinolone consumption data from three Italian regions. Drug Saf. 26 (2), 109–120. 10.2165/00002018-200326020-00004 12534327

[B19] Lertxundi U. Palacios R. H. Gutierrez F. C. Echaburu S. D. Garcia M. G. Gomez C. A. (2013). Levofloxacin-induced delirium in a patient suffering from schizoaffective disorder and multiple sclerosis. Curr. Drug Saf. 23914753

[B20] Maharani B. Jafrin A. L. Bai K. V. Sivagnanam G. (2019). Levofloxacin-induced tactile hallucination and acute anxiety reaction. Indian J. Pharmacol. 51 (2), 123–125. 10.4103/ijp.IJP_291_17 31142949 PMC6533930

[B21] Mahoney M. V. Swords K. E. (2021). Fluoroquinolones: friends or foes? Clin. Infect. Dis. 73 (5), 857–858. 10.1093/cid/ciab150 34492696

[B22] Mattappalil A. Mergenhagen K. A. (2014). Neurotoxicity with antimicrobials in the elderly: a review. Clin. Ther. 36 (11), 1489–1511. 10.1016/j.clinthera.2014.09.020 25450476

[B23] Miczek K. A. Weerts E. M. Vivian J. A. Barros H. M. (1995). Aggression, anxiety and vocalizations in animals: GABAA and 5-HT anxiolytics. Psychopharmacology 121 (1), 38–56. 10.1007/BF02245590 8539340

[B24] Muanda F. T. Sood M. M. Weir M. A. Sontrop J. M. Ahmadi F. Yoo E. (2022). Association of higher-dose fluoroquinolone therapy with serious adverse events in older adults with advanced chronic kidney disease. JAMA Netw. Open 5 (8), e2224892. 10.1001/jamanetworkopen.2022.24892 35917124 PMC9346548

[B25] Munjal S. Smolin Y. (2017). Ciprofloxacin associated exacerbation of anxiety in an elderly patient: a review of anxiogenic potential of fluoroquinolones. J. Clin. Psychopharmacol. 37 (3), 370–372. 10.1097/JCP.0000000000000696 28306617

[B26] Pozo E. D. Arana-Asensio E. Garcia-Lopez P. (2015). Acute confusional syndrome induced by moxifloxacin in an elderly man. J. Am. Geriatr. Soc. 63 (12), 2647–2648. 10.1111/jgs.13856 26691713

[B27] Ruan S. Tu C. H. Bourne C. R. (2024). Friend or foe: protein inhibitors of DNA gyrase. Biology-Basel 13 (2), 84. 10.3390/biology13020084 38392303 PMC10886550

[B28] Sanders V. R. Sweeney A. Topf M. Millar N. S. (2022). Stoichiometry-selective antagonism of α4β2 nicotinic acetylcholine receptors by fluoroquinolone antibiotics. ACS Chem. Neurosci. 13 (12), 1805–1817. 10.1021/acschemneuro.2c00200 35657695 PMC9204775

[B29] Sellick J. Mergenhagen K. Morris L. Feuz L. Horey A. Risbood V. (2018). Fluoroquinolone-related neuropsychiatric events in hospitalized veterans. Psychosomatics 59 (3), 259–266. 10.1016/j.psym.2017.11.001 29275962

[B30] Sen S. Jaiswal A. K. Yanpallewar S. Acharya S. B. (2007). Anxiogenic potential of ciprofloxacin and norfloxacin in rats. Singap. Med. J. 48 (11), 1028–1032. 17975693

[B31] Smelt C. Sanders V. R. Newcombe J. Burt R. P. Sheppard T. D. Topf M. (2018). Identification by virtual screening and functional characterisation of novel positive and negative allosteric modulators of the α7 nicotinic acetylcholine receptor. Neuropharmacology 139, 194–204. 10.1016/j.neuropharm.2018.07.009 30009834 PMC6078708

[B32] Tian D. Shi W. Yu Y. Zhou W. Tang Y. Zhang W. (2023). Enrofloxacin exposure induces anxiety-like behavioral responses in zebrafish by affecting the microbiota-gut-brain axis. Sci. Total Environ. 858 (Pt 3), 160094. 10.1016/j.scitotenv.2022.160094 36372168

[B33] Wierzbinski P. Hubska J. Henzler M. Kucharski B. Bies R. Krzystanek M. (2023). Depressive and other adverse CNS effects of fluoroquinolones. Pharmaceuticals 16 (8), 1105. 10.3390/ph16081105 37631020 PMC10459424

[B34] Zareifopoulos N. Panayiotakopoulos G. (2017). Neuropsychiatric effects of antimicrobial agents. Clin. Drug Invest. 37 (5), 423–437. 10.1007/s40261-017-0498-z 28197902

[B35] Zhou C. Peng S. Lin A. Jiang A. Peng Y. Gu T. (2023). Psychiatric disorders associated with immune checkpoint inhibitors: a pharmacovigilance analysis of the FDA Adverse Event Reporting System (FAERS) database. EClinicalMedicine 59, 101967. 10.1016/j.eclinm.2023.101967 37131541 PMC10149185

