# Supplementary figures and images for: Psychiatric disorders associated with fluoroquinolones: a pharmacovigilance analysis of the FDA adverse event reporting system database
Source: Front Pharmacol. 2024 Oct 14;15:1435923. doi: 10.3389/fphar.2024.1435923 (PMC11513374; doi:10.3389/fphar.2024.1435923)

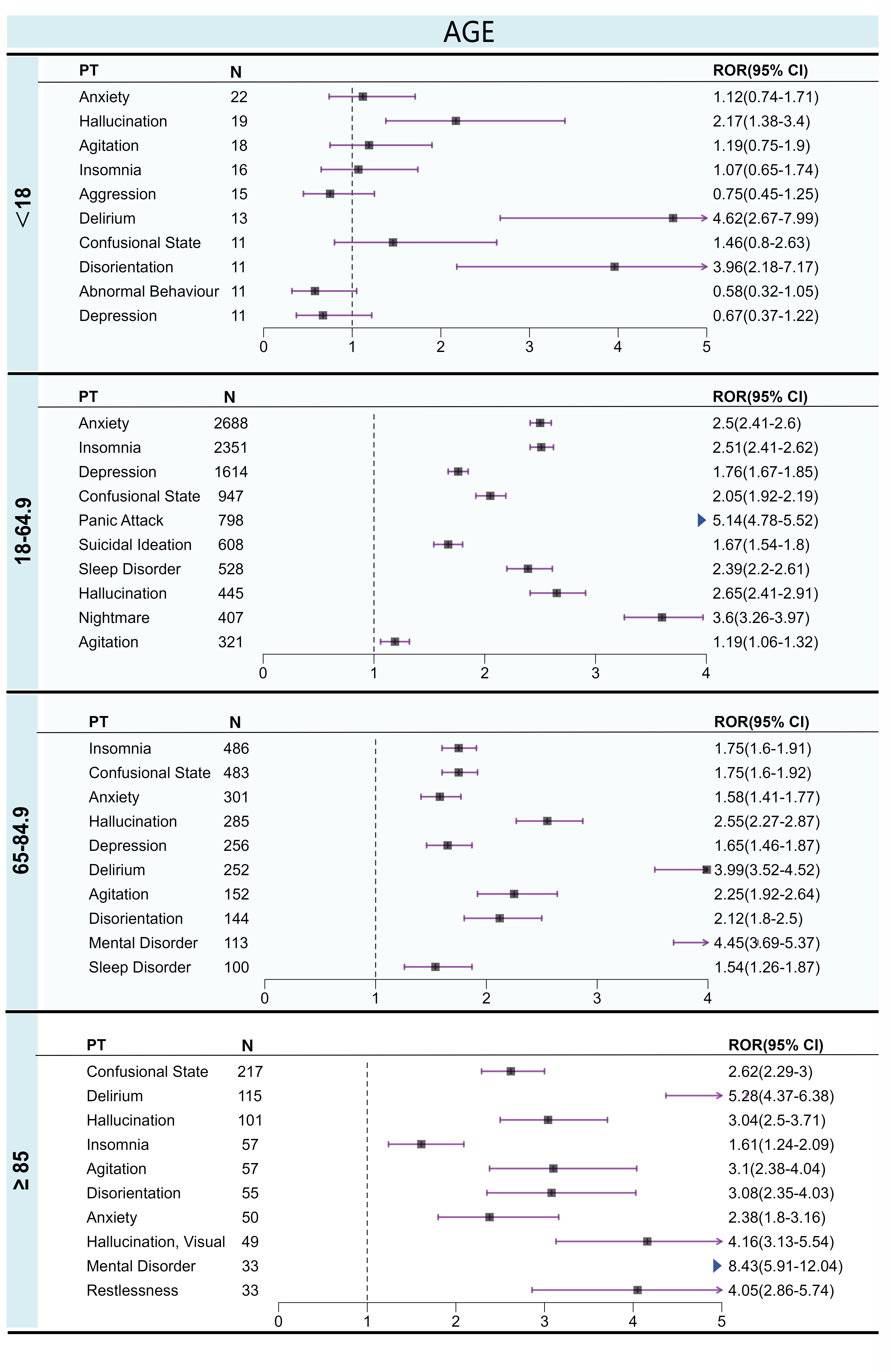

Supplement: Supplementary file 2 [file Image3.JPEG]

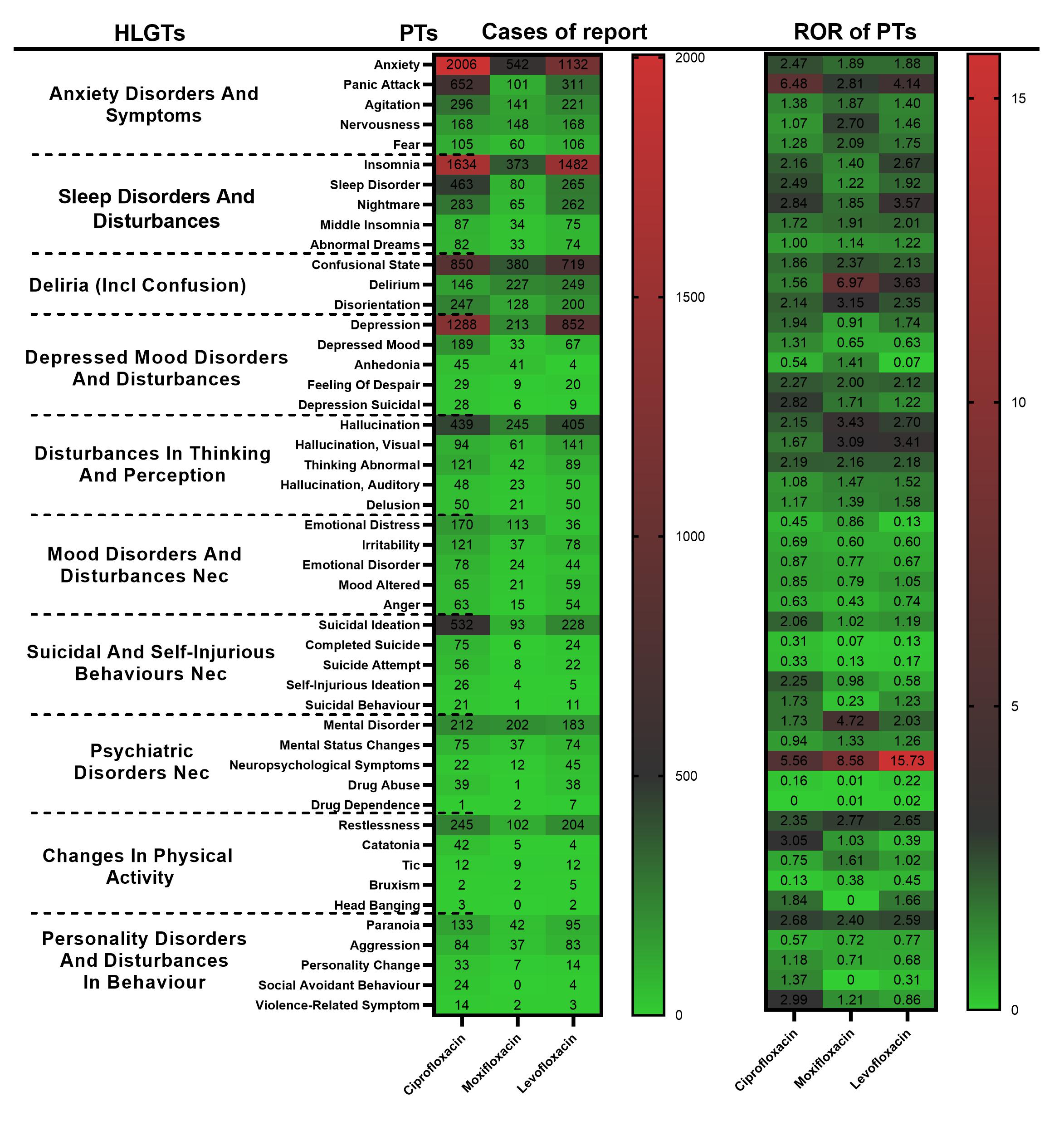

Supplement: Supplementary file 5 [file Image1.JPEG]

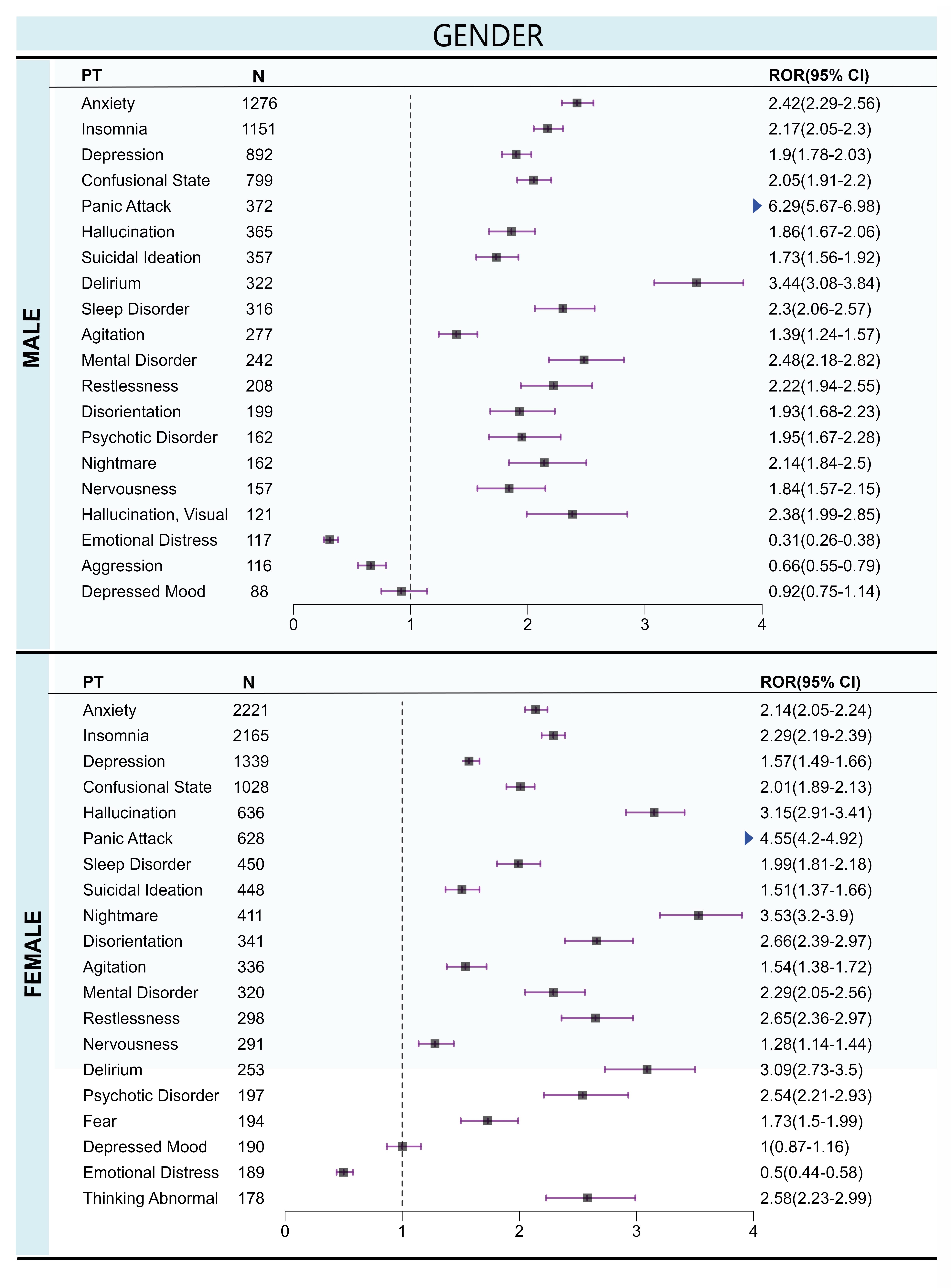

Supplement: Supplementary file 6 [file Image2.JPEG]
