# Supplementary material for: Psychiatric disorders associated with fluoroquinolones: a pharmacovigilance analysis of the FDA adverse event reporting system database
Source: Front Pharmacol. 2024 Oct 14;15:1435923. doi: 10.3389/fphar.2024.1435923 (PMC11513374; doi:10.3389/fphar.2024.1435923)
Supplement: Supplementary file 4 [file Table1.DOCX]

Supplementary Material

**Supplementary Table 1. Algorithm for disproportionate**

| **Drugs** | **Target AE cases** | **All other AE cases** |
| --- | --- | --- |
| Target drug | a | b |
| All other drug | c | d |

| **Method** | **Formula** | **Requirements** |
| --- | --- | --- |
| ROR | $ROR=(a/c)(b/d)=ad/bc$ | a≥3, 95% CI (lower limit)＞1 |
|  | $95\%CI=e^{ln(ROR)\pm1.96\sqrt{(\frac{1}{a}+\frac{1}{b}+\frac{1}{c}+\frac{1}{d})}}$ |  |
| BCPNN | $IC=\log_{2}\frac{a(a+b+c+d)}{(a+b)(a+c)}$ $E(IC)=\log_{2}\frac{(a+\gamma11)(a+b+c+d+\alpha)(a+b+c+d+\beta)}{(a+b+c+d+\gamma)(a+b+a1)(a+c+\beta1)} V$  $(IC)=\frac{1}{({ln2)}^{2}}\left\{ \left[ \frac{(a+b+c+d)-a+\gamma-\gamma11}{(a+\gamma11)(1+a+b+c+d+\gamma)} \right]+\left[ \frac{(a+b+c+d)-(a+b)+\alpha-\alpha1}{(a+b+\alpha1)(1+a+b+c+d+\alpha)} \right]+\left[ \frac{(a+b+c+d)-(a+c)+\beta-\beta1}{(a+c+\beta1)(1+a+b+c+d+\beta)} \right] \right\}$  $\gamma=\gamma11\frac{(a+b+c+d+\alpha)(a+b+c+d+\beta)}{(a+b+\alpha1)(a+c+\beta1)}$ $IC­2SD=E(IC)-2\sqrt{V(IC)}$ | IC025＞0 |

**Supplementary table 2.** Results of the BCPNN method for analyzing PT after SMQ clustering.

**Supplementary table 3.** Results of the BCPNN method for analyzing SMQs for three fluoroquinolones.

**Supplementary Figure 1.** Signal strength of HLGTs-PTs ADR for three fluoroquinolones belonging to psychiatric disorders.

**Supplementary Figure 2.** Top 20 fluoroquinolone-related psychiatric ADRs reported by Female and Male.

**Supplementary Figure 3.** Top 10 fluoroquinolone-related psychiatric ADRs reported in different age groups.
